# Polymorphism of regulatory region of APEH gene (c.-521G>C, rs4855883) as a relevant predictive factor for radiotherapy induced oral mucositis and overall survival in head neck cancer patients

**DOI:** 10.18632/oncotarget.25662

**Published:** 2018-07-03

**Authors:** Anna Brzozowska, Radosław Mlak, Iwona Homa-Mlak, Paweł Gołębiowski, Marcin Mazurek, Marzanna Ciesielka, Teresa Małecka-Massalska

**Affiliations:** ^1^ Department of Oncology, Medical University of Lublin, Lublin 20-090, Poland; ^2^ Department of Human Physiology, Medical University of Lublin, Lublin 20-080, Poland; ^3^ Department of Forensic Medicine, Medical University of Lublin, Lublin 20-090, Poland

**Keywords:** oral mucositis, survival, radiotherapy, head and neck cancer, polymorphism

## Abstract

**Background:**

The study purpose was to examine the correlation between SNP in the regulatory region (c.-521G>C, rs4855883) of *APEH* gene as well as the incidence and severity of radiotherapy (RTH) induced oral mucositis (OM) and overall survival (OS) in head and neck cancer (HNC) patients.

**Methods:**

OM in 62 HNC patients subjected to irradiation was assessed using RTOG/EORTC scale. DNA was isolated from whole blood of HNC patients. Mini-sequencing method (SNaPshot PCR) was used to determine the genotype.

**Results:**

The following frequency of occurrence of *APEH* gene was observed: CC: 37.1%, CG: 43.6% and GG: 19.3%. It was established that the presence of CC genotype reduced the risk of occurrence of grade 2 and 3 OM symptoms: 3-fold in RTH week 2 (in case of CC vs GC or GG it was: 26.8% vs 73.2% patients, respectively, OR = 0.27, 95 CI: 0.09–0.83; *p* = 0.0222), 6-fold in RTH week 3 (in case of CC vs GC or GG it was: 29.4% vs 70.6% patients, respectively, OR = 0.16, 95 CI: 0.04–0.67; *p* = 0.0125) and grade 3 OM symptoms 4-fold in RTH week 6 (in case of CC vs GC or GG it was: 19.2% vs 80.8% patients, respectively, OR = 0.23, 95 CI: 0.07–0.77; *p* = 0.0166). CC genotype was associated with lower OS (CC vs GG or GC: 29 months vs 38 months; HR = 2.48, 95% CI: 0.90–6.85; *p* = 0.0266).

**Conclusion:**

CC genotype of *APEH* gene was correlated with the risk of more severe radiotherapy-induced OM in HNC patients and lower rates of survival.

## INTRODUCTION

One of the most common cancers are epithelial tumors of the head and neck area (HNC) and the number of new cases reaches 650,000 cases per year [1]. Radical methods of HNC treatment (surgical, radiotherapy [RTH], chemotherapy [CTH]) allow to achieve good results in terms of overall and disease-free survival. However, they are also associated with high toxicity. During radical RTH, almost 80% of patients develop acute radiation-induced reaction (OM), especially with simultaneous CTH [2–5]. OM is clinically manifested by inflammation. It develops gradually leading to severe pain, ulceration and dysphagia. In 11% patients, the severity of symptoms requires hospitalization and intensive treatment [3, 5]. Severe OM often results in discontinuation of RTH, which increases the risk of non-treatment, because every 5 days of interruption in RTH increases the risk of progression by 14% [2, 3]. Severe OM, 3 and 4 stages according to the RTOG/EORTC (Radiation Therapy Oncology Group/ European Organisation for Research and Treatment of Cancer) scale is clinically significant [2]. Its occurrence is individually variable. Despite the known risk factors such as older age, male gender, oral hygiene, total radiation dose, smoking, systemic diseases, RTH technique and combined chemoradiation, there is still no clinical tool in the daily practice to assess the risk of OM severity in patients with severe HNC subjected to radical RTH [4]. Radiation induced OM is a gradual and multi-stage process in which the key and initiating role is played by free radicals (reactive oxygen species, ROS) [2]. After exposure to RTH, macromolecules such as DNA and membrane lipids are damaged [2]. However, the main effect of RTH is based on ionization of water atoms, which results in the occurrence of ROS including superoxide radical (O_2_°–), hydroxyl radical (OH°), and hydrogen peroxide (H_2_O_2_). ROS damage cell elements and activate the nuclear factor kappa-light-chain-enhancer of activated B cells (NF-κB) leading to higher transcription of genes for proinflammatory cytokines: IL-6, IL-1B and TNF-α (tumor necrosis factor α). There occurs tissue damage and activation of apoptosis [2]. DNA breakage and ROS stimulate the formation of sphingomyelinase and/or ceramide synthase, which leads to the activation of ceramide pathway resulting in apoptosis. DNA breakage and ROS also lead to the breakup of fibronectin activating macrophages and Matrix Metalloproteinases (MMPs). They also promote apoptosis [2]. The process of ROS detoxification involves many factors. Recent studies indicate an important role of acylpeptidehydrolase (APEH) in this process. It was first identified as oxidized protein hydrolase (OPH) [6]. It has been hypothesized that APEH is involved in ROS detoxification as one of phase 3 antioxidant enzymes. They participate in the elimination of proteins that have been irreversibly denatured [6, 7].

APEH is a 75–80 kDa peptidase and one of the four members of prolyl oligopeptidase family of serine proteases. APEH degrades 30 to 50 amino acid long peptides and preferentially degrades oxidized peptides [9]. The precise biological activity of APEH has not been determined [8]. APEH has been reported to regulate the activity of proteasome [10]. APEH may provide an alternative mechanism for proteasome degradation [11].

Despite the data confirming that the risk of radiotoxicity depends on genetic instability, so far few studies have evaluated the relationship between different genes and their polymorphisms (including single nucleotide polymorphisms, SNPs) and the severity of OM in HNC patients [12–18]. However, there is no *APEH* gene study concerning of RTH toxicity assessment.

The hypothesis of our study assumes that the severity of OM in irradiated patients with HNC may depend on the status of the *APEH* gene, including the SNPs present in it, which may potentially modify the expression or functioning of its protein product.

The aim of the study was to assess the *APEH* gene polymorphism and to analyze the relationship between their occurrence and OM severity and overall survival (OS) in patients with HNC.

## RESULTS

There were approximately 82% men and 18% women in the study group. The median age was 63 years. 91.9% patients were diagnosed with squamous-cell carcinoma. 96.8% patients had advanced tumor (stages III–IV) at the time of enrollment. 72.6% patients in the study group underwent surgery followed by RTH or chemoradiation, 4.84% were treated using neoadjuvant CTH followed by RTH, 11.3% were treated using RTH alone and 11.3% were treated by concurrent chemoradiation. All enrolled patients were subjected to RTH. 93.5% patients received complete dose and in case of the remaining subjects treatment was discontinued due to deterioration of their general health status. 45.2% patients declared excessive alcohol consumption. Large majority of patients who have ever smoked (86.5%) were current tobacco smokers. Detailed patient characteristics is presented in Table 1.

**Table 1:** Characteristics of the study group

Factor		Study group (*n* = 62)
Gender	Male	51 (82.25%)
	Female	11 (17.75%)
Age, median (range)		63 (42–87)
Histopathological diagnosis	Squamous cell carcinoma	57 (91.94%)
	Other	5 (8.06%)
T stage	T1	2 (3.23%)
	T2	9 (14.52%)
	T3	15 (24.19%)
	T4	36 (58.06%)
N stage	Nx	2 (3.23%)
	N0	18 (29.03%)
	N1	6 (9.68%)
	N2	32 (51.61%)
	N3	4 (6.45%)
M stage	Mx	3 (75.00%)
	M1	1 (25.00%)
Overall stage	I	2 (3.23%)
	III	12 (19.35%)
	IVA	40 (64.52%)
	IVB	3 (4.84%)
	IVC	5 (8.06%)
Type of treatment	Surgery + RTH	28 (45.16%)
	Surgery + chemoradiation	17 (27.42%)
	RTH alone	7 (11.29%)
	Induction CHTH + RTH	3 (4.84%)
	Concurrent chemoradiation	7 (11.29%)
Alcohol consumption	Yes	28 (45.16%)
	No	34 (54.84%)
Tobacco smoking	Yes	52 (83.87%)
	No	10 (16.13%)
Current smoking	Yes	45 (86.54%)
	No	7 (13.46%)

The following frequency of occurrence of *APEH* gene (c.-521G>C, rs4855883) genotypes was observed: CC: 37.1%, CG: 43.6% and GG: 19.3%. Genotype frequencies are similar to those in dbSNP database (frequency of CC, CG i GG in the database is 32%, 54% and 14% respectively). The examined genotypes retained the Hardy-Weinberg equilibrium (*p* = 0.3199). The genotype distribution was not correlated with the demographic and clinical factors. The influence of the demographic-clinical factors on the genotype distribution was presented in Table 2.

**Table 2:** Influence of demographic and clinical factors on distribution of APEH genotype

Factor		*APEH* genotypes			
		CC (*n* = 23; 7.1%)	GG (*n* = 12; 9.3%)	CG (*n* = 27; 3.6%)	*p* χ^2^
Gender	Male	21 (41.18%)	11 (21.57%)	19 (37.25%)	0.0987 4.632
	Female	2 (18.19%)	1 (9.10%)	8 (72.73%)	
Age (years)	≥63	16 (47.06%)	4 (11.76%)	14 (41.18%)	0.1135 4.352
	<63	7 (25.00%)	8 (28.57%)	13 (46.43%)	
Histopathological diagnosis	Squamous cell carcinoma	22 (38.60%)	12 (21.05%)	23 (40.35%)	0.2080 3.141
	Other	1 (20.00%)	-	4 (80.00%)	
Stage of disease (TNM classification)	I	-	-	2 (100.00%)	0.6736 5.764
	III	4 (33.33%)	2 (16.67%)	6 (50.00%)	
	IVA	14 (35.00%)	9 (22.5%)	17 (42.5%)	
	IVB	2 (66.67%)	-	1 (33.33%)	
	IVC	3 (60.00%)	1 (20.00%)	1 (20.00%)	
Type of treatment	Surgery + RTH	13 (46.43%)	4 (14.29%)	11 (39.29%)	0.1160 12.882
	Surgery + chemoradiation	3 (17.65%)	7 (41.18%)	7 (41.18%)	
	RTH alone	4 (57.14%)	-	3 (42.86%)	
	Induction CHTH + RTH	2 (66.67%)	-	1 (33.33%)	
	Concurrent chemoradiation	1 (14.29%)	1 (14.29%)	5 (71.43%)	
Anemia before the treatment	Yes	4 (30.77%)	1 (7.69%)	8 (61.54%)	0.2786 2.556
	No	19 (38.77)	11(22.45%)	19 (38.77)	
Alcohol consumption	Yes	9 (32.14%)	5 (17.86%)	14 (50.00%)	0.6424 0.885
	No	14 (41.18%)	7 (20.59%)	13 (38.24%)	
Tobacco smoking	Yes	20 (38.46%)	8 (15.39%)	24 (46.15%)	0.1930 3.290
	No	3 (30.00%)	4 (40.00%)	3 (30.00%)	
Current smoking	Yes	17 (37.78%)	8 (17.78%)	20 (44.44%)	0.8774 0.262
	No	6 (35.29%)	4 (23.53%)	7 (41.18%)	

The influence of demographic and clinical factors on the intensity of OM was assessed. No significant alterations were found in the severity of OM in week 6 in relation to the occurrence of respective demographic and clinical factors. The only exceptions were: treatment involving surgery followed by RTH (lower risk of OM grade 3 - OM grade 1 and 2 vs 3: 78.57% vs 21.43%; HR = 0.19, 95% CI: 0.06–0.59; *p* = 0.0041) and surgery followed by chemoradiation (higher risk of OM grade 3 - OM grade 1 and 2 vs 3: 35.29% vs 64.71%; HR = 3.67, 95% CI: 1.14–11.84; *p* = 0.0298). The effects of the influence of demographic and clinical factors on OM severity were presented in detail in Table 3.

**Table 3:** Influence of demographic and clinical factors on the severity of oral mucositis after 6th week of radiotherapy

Factor			Radiation reaction grade in 6th week of RTH					
			1 (*n* = 5; 8.1%)	2 and 3 (*n* = 57; 91.9%)	*P* OR [95% CI]	1 and 2 (*n* = 36; 58.1%)	3 (*n* = 26; 41.9%)	*P* OR [95% CI]
Gender	Male		5 (%)	46 (%)	0.5084 0.37 [0.02–7.14]	30(58.82%)	21 (41.18%)	0.7944 0.84 [0.23–3.12]
	Female		–	11 (100%)		6 (54.55%)	5 (45.45%)	
Age (years)	≥63		5 (14.7%)	29 (85.30%)	0.1000 0.08 [0.01–1.60]	22(64.70%)	12(35.30%)	0.2448 0.54 [0.20–1.51]
	<63		–	28 (100%)		14(50.0%)	14(50.0%)	
Histopathological diagnosis	Squamous cell carcinoma		5 (8.77%)	52 (91.23%)	0.9268 0.87 [0.04–17.88]	35 (61.40%)	22 (38.60%)	0.1078 0.16 [0.02–1.50]
	Other		–	5 (100%)		1 (20.00%)	4 (80.00%)	
Stage of disease (TNM classification)	I– III		2 (14.29%)	12 (85.71%)	0.3443 0.40 [0.06–2.67]	9 (64.29%)	5 (35.71%)	0.5928 0.71 [0.21–2.45]
	IV		3 (6.25%)	45 (93.75%)		27 (56.25%)	21 (43.75%)	
Type of treatment	Surgery + RTH	Yes	3 (10.71%)	25 (89.29%)	0.4928 0.52 [0.08–3.36]	22 (78.57%)	6 (21.43%)	0.0041 0.19 [0.06–0.59]
		Other	2 (5.88%)	32 (94.12%)		14 (41.18%)	20 (58.82%)	
	Surgery + chemoradiation	Yes	–	17 (100.00%)	0.3002 4.75 [0.25–90.71]	6 (35.29%)	11 (64.71%)	0.0298 3.67 [1.14–11.84]
		Other	5 (11.11%)	40 (88.89%)		30 (66.67%)	15 (33.33%)	
	RTH alone	Yes	2 (28.57%)	5 (71.43%)	0.0591 0.14 [0.02–1.10]	4 (57.14%)	3 (42.86%)	0.9582 1.04 [0.21–5.12]
		Other	3 (5.45%)	52 (94.55%)		32 (58.18%)	23 (41.82%)	
	Induction CHTH + RTH	Yes	–	3 (100.00%)	0.8255 0.71 [0.03–15.53]	2 (66.67%)	1 (33.33%)	0.7582 0.68 [0.06–7.72]
		Other	5 (8.47%)	54 (91.53%)		34 (40.68%)	25(42.37%)	
	Concurrent chemoradiation	Yes	–	7 (100.00%)	0.7481 1.63 [0.08–32.65]	2 (28.57%)	5 (71.43%)	0.1127 4.05 [0.72–22.78]
		Other	5 (9.10%)	50 (90.90%)		34 (61.82%)	21 (38.18%)	
Anemia before the treatment	Yes (*n* = 13; 21%)		–	13 (1005)	0.4246 3.34 (0.17–64.30)	7 (53.85%)	6 (46.15%)	0.7291 1.24 (0.36–4.25)
	No (*n* = 49; 79%)		5 (8.47%)	44 (91.53%)		29 (59.18%)	20 (40.82%)	
Alcohol consumption	Yes (*n* = 28; 45.2%)		1 (3.57%)	27 (96.43%)	0.2650 3.60 [0.38–34.23]	16 (57.14%)	12 (42.86%)	0.8938 1.07 [0.39–2.95]
	No (*n* = 34; 54.8%)		4 (11.76%)	30 (88.24%)		20 (58.82%)	14 (41.18%)	
Tobacco smoking	Yes (*n* = 52; 83.9%)		4 (7.70%)	48 (92.30%)	0.8067 1.33 [0.13–13.35]	31 (59.62%)	21 (40.38%)	0.5740 0.68 [0.17–2.63]
	No (*n* = 10; 16.1%)		1 (10%)	9 (90%)		5 (50.00%)	5 (50.00%)	
Current smoking	Yes (*n* = 45; 72.6%)		4 (8.89%)	41 (91.11%)	0.6406 0.64 [0.07–6.18]	27 (60.00%)	18 (40.00%)	0.6158 0.75 [0.24–2.31]
	No (*n* = 17; 27.4%)		1 (5.88%)	16 (94.12%)		9 (52.95%)	8 (47.05%)	

It was established that the presence of CC genotype reduced the risk of occurrence of grade 2 and 3 OM symptoms 3-fold in RTH week 2 (in case of CC vs GC or GG it was: 26.8% vs 73.2% patients, respectively, OR = 0.27, 95 CI: 0.09–0.83; *p* = 0.0222). Similarly, CC genotype reduced the risk of occurrence of grade 2 and 3 OM symptoms 6 -fold in week 3 of RTH (in case of CC vs GC or GG it was: 29.4% vs 70.6% patients, respectively, OR = 0.16, 95 CI: 0.04–0.67; *p* = 0.0125). Moreover, CC genotype reduced the risk of occurrence of grade 3 OM symptoms 4 -fold in week 6 of RTH (in case of CC vs GC or GG it was: 19.2% vs 80.8% patients, respectively, OR = 0.23, 95 CI: 0.07–0.77; *p* = 0.0166).

Detailed results regarding the scope of OM progression after consecutive courses of RTH depending on the presence of specific *APEH* gene genotypes are shown in Table 4.

**Table 4:** The influence of APEH genotypes on the severity of oral mucositis after subsequent weeks of radiotherapy

RTH week	Radiation reaction grade	CC (*n* = 23; %)	GG or GC (*n* = 39; %)	*p*, OR [95% CI]	GG (*n* = 12; %)	CC or GC (*n* = 50; %)	*p*, OR [95% CI]	CG (*n* = 27; %)	CC or GG (*n* = 35; %)	*p*, OR [95% CI]
**1**	**0** (*n* = 6; 9.7%)	4 (66.67%)	2 (33.33%)	0.1355 0.26 [0.04–1.53]	–	6 (100.0%)	0.3886 3.65 [0.19–69.36]	2 (33.33%)	4 (66.67%)	0.5981 1.61 [0.27–9.54]
	**1** (*n* = 56; 90.3%)	19 (32.20%)	37 (67.80%)		12 (21.43%)	44 (78.57%)		25 (44.64%)	31 (55.36%)	
**2**	**1** (*n* = 36; 58.1%)	16 (44.44%)	20 (55.56%)	0.1624 0.46 [0.15–1.37]	7 (19.44%)	29 (80.56%)	0.9832 0.99 [0.27–3.54]	13 (36.11%)	23 (63.89%)	0.1671 2.06 [0.74–5.77]
	**2** (*n* = 26; 41.9%)	7 (26.92%)	19 (73.08%)		5 (19.23%)	21 (80.77%)		14 (53.85%)	12 (33.33%)	
**3**	**1** (*n* = 21; 33.9%)	12 (57.14%)	9 (42.86%)	0.0222 0.27 [0.09–0.83]	3 (14.29%)	18 (185.71%)	0.4728 1.69 [0.40–7.04]	6 (28.57%)	15 (71.43%)	0.0934 2.08 [0.68–6.36]
	**2 and 3** (*n* = 41; 66.1%)	11 (26.83%)	30 (73.17%)		9 (21.95%)	32 (78.05%)		21 (51.22%)	20 (48.78%)	
	**1 and 2** (*n* = 56; 90.3%)	22 (39.29%)	34 (60.71%)	0.2984 0.31 [0.03–2.83]	11 (19.64%)	45 (26.79%)	0.8610 0.82 [0.09–7.73]	23 (41.07%)	33 (58.93%)	0.1810 2.62 [0.85–8.11]
	**3** (*n* = 6; 9.7%)	1 (16.67%)	5 (83.33%)		1 (16.67%)	5 (83.33%)		4 (66.67%)	2 (33.33%)	
**4**	**1** (*n* = 11; 17.7%)	8 (72.73%)	3 (27.27%)	0.0125 0.16 [0.04–0.67]	2 (18.18%)	9 (81.82%)	0.9136 1.10 [0.20–5.89]	1 (9.09%)	10 (90.91%)	0.0310 10.40 [1.23–87.31]
	**2 and 3** (*n* = 51; 82.3%)	15 (29.41%)	36 (70.59%)		10 (19.60%)	41 (80.40%)		26 (50.98%)	25 (49.02%)	
	**1 and 2** (*n* = 48; 77.4%)	20 (41.67%)	28 (58.33%)	0.1776 0.38 [0.09–1.55]	7 (14.58%)	41 (85.42%)	0.0880 3.25 [0.84–12.62]	21 (43.75%)	27 (56.25%)	0.9527 0.96 [0.29–3.21]
	3 (*n* = 14; 22.6%)	3 (21.43%)	11 (78.57%)		5 (35.71%)	9 (64.29%)		6 (42.86%)	8 (57.14%)	
**5**	1 (*n* = 6;9.7%)	4 (66.67%)	2 (33.33%)	0.1355 0.26 [0.04–1.53]	2 (33.33%)	4 (66.67%)	0.3723 0.43 [0.07–2.71]	–	6 (100.00%)	0.0944 12.12 [0.65–225.38]
	2 and 3 (*n* = 56.;90.3%)	19 (33.93%)	37 (66.07%)		10 (17.86%)	46 (82.14%)		27 (48.21%)	29 (51.79%)	
	1 and 2 (*n* = 48;77.4%)	20 (41.67%)	28 (58.33%)	0.1776 0.38 [0.09–1.55]	7 (14.58%)	41 (85.42%)	0.0880 3.25 [0.84–12.62]	21 (43.75%)	27 (56.25%)	0.9527 0.96 [0.29–3.21]
	3 (*n* = 14; 22.6%)	3 (21.43%)	11 (78.57%)		5 (35.71%)	9 (64.29%)		6 (42.86%)	8 (57.14%)	
**6**	1 (*n* = 6; 9.7%)	4 (66.67%)	2 (33.33%)	0.1355 0.26 [0.04–1.53]	2 (33.33%)	4 (66.67%)	0.3723 0.43 [0.07–2.71]	–	6 (100.00%)	0.0944 12.12 [0.65–225.38]
	**2 and 3** (*n* = 56; 90.3%)	19 (33.93%)	37 (66.07%)		10 (17.86%)	46 (82.14%)		27 (48.21%)	29 (51.79%)	
	**1 and 2** (*n* = 47; 75.8%)	18 (38.30%)	29 (61.70%)	0.7292 0.81 [0.24–2.74]	10 (21.28%)	37 (78.72%)	0.5018 0.57 [0.11–2.95]	19 (40.43%)	28 (59.57%)	0.3824 1.68 [0.52–5.42]
	**3** (*n* = 15; 24.2%)	5 (33.33%)	10 (66.67%)		2 (13.33%)	13 (86.67%)		8 (53.33%)	7 (46.67%)	
**7**	**1** (*n* = 5; 8.1%)	4 (80.00%)	1 (20.00%)	0.0713 0.12 [0.01–1.20]	1 (20.00%)	4 (80.00%)	0.9696 0.96 [0.10–9.43]	–	5 (100.00%)	0.1262 9.92 [0.52–187.73]
	**2 and 3** (*n* = 57; 91.9%)	19 (33.33%)	38 (66.67%)		11 (19.30%)	46 (80.70%)		27 (47.37%)	30 (52.63%)	
	**1 and 2** (*n* = 36; 58.1%)	18 (50.00%)	18 (50.00%)	0.0166 0.23 [0.07–0.77]	4 (11.11%)	32 (88.89%)	0.0619 3.56 [0.94–13.47]	14 (38.89%)	22 (61.11%)	0.3850 1.57 [0.57–4.36]
	**3** (*n* = 26; 41.9%)	5 (19.23%)	21 (80.77%)		8 (30.77%)	18 (69.23%)		13 (50.00%)	13 (50.00%)	

**Figure 1:**
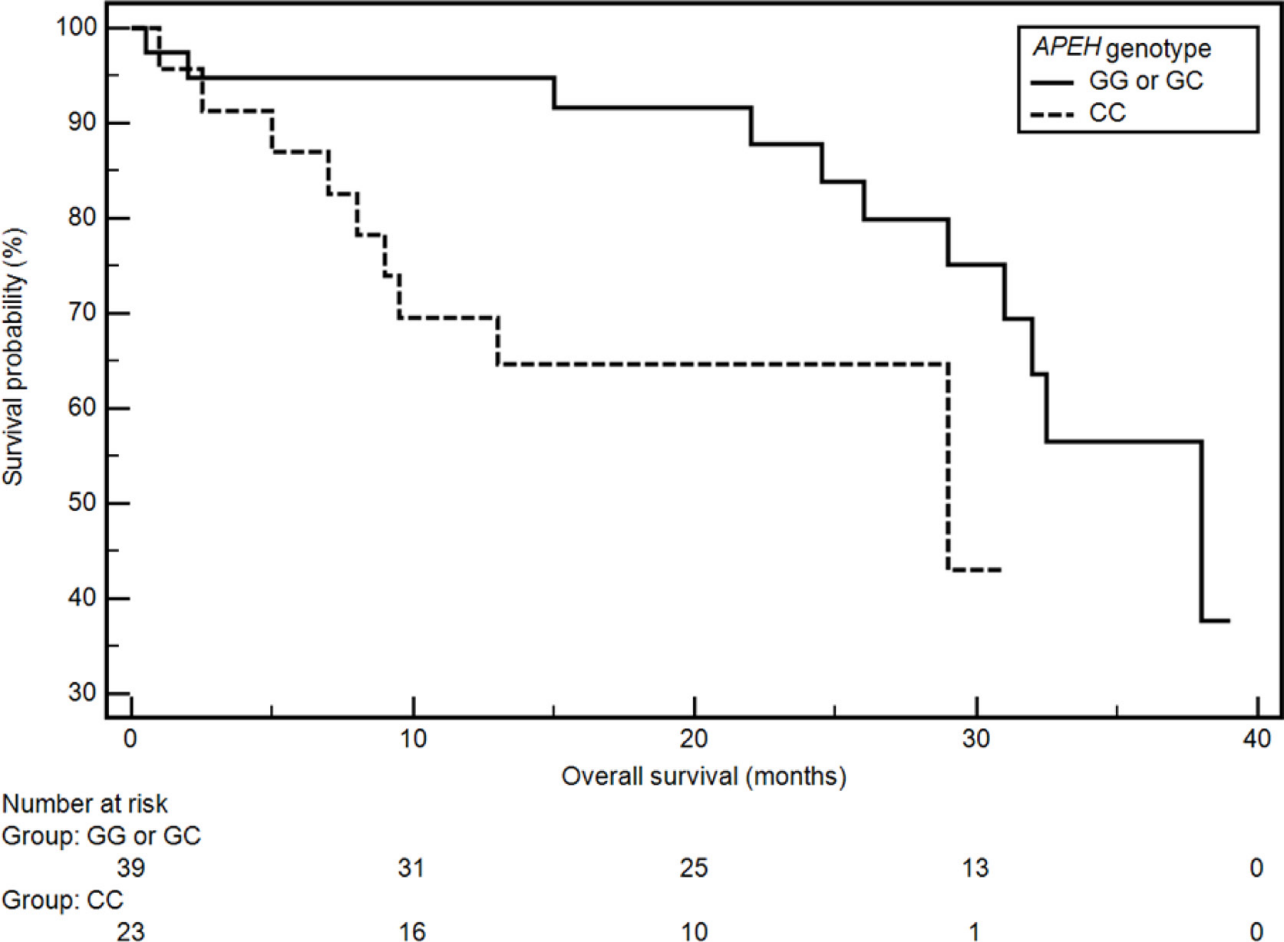
The probability of overall survival alteration depending on APEH genotype

CC genotype of *APEH* gene was significantly correlated with an increased risk of lower OS (CC vs GG or GC: 29 months vs 38 months; HR = 2.48, 95% CI: 0.90–6.85; *p* = 0.0266; Figure 1). On the basis of Cox regression analysis (after adjustment for gender, age, histopathological diagnosis, stage of disease - TNM classification anemia before the treatment and tobacco smoking status) we found that CC genotype of *APEH* gene was an independent prognostic factor. The results of Cox analysis (overall model fit: χ^2^ = 7.09, *p* = 0.4198) are presented in Table 5.

**Table 5:** Multivariable Cox regression analysis results

Covariate	HR	95% CI	*p*
*APEH* genotype (CC)	3.04	1.07–8.63	0.0381
Age (>63)	0.95	0.32–2.77	0.9242
Gender (male)	0.70	0.18–2.70	0.6038
Histopathological diagnosis (non-squamous)	1.22	0.21–7.12	0.8221
Stage (IV)	1.02	0.31–3.36	0.9688
Anemia before the treatment	0.01	0.25–3.54	0.9238
Current smoking (yes)	2.50	0.69–9.05	0.1634

## DISCUSSION

Severe OM occurring in some patients with HNC during radical RTH is a serious problem in everyday clinical practice. It leads to considerable symptoms, often limiting the planned treatment. OM is characterized by highly individual variability and despite the known risk factors (older age, male gender, oral hygiene, total radiation dose, smoking, systemic diseases, RTH technique and combined chemoradiation) we are not able to fully predict the response of tissues to irradiation in individual patients.

This variability of individuals may result from genetic differences leading to differences in radiosensitivity of tissues to ionizing radiation. The most common genetic change are SNPs, defined as polymorphisms in which the minor variant (allele) is present in at least 1% of a given population [13, 14].

The association between normal tissue complications after RTH and polymorphic variations in *TGFB1* and *XRCC1* genes was described by Alsbeih *et al.* [19]. In turn, Pratesi *et al.* assessed the association between OM in HNC patients and SNPs of *XRCC1* (c.1196A>G) (allele A) and *RAD51* (c.-3429G>C) (allele C) genes [16]. Yu *et al.* demonstrated that genetic polymorphisms of Wnt/β-catenin pathway genes are associated with the efficacy and toxicities of RTH in patients with nasopharyngeal carcinoma [20]. Le *et al.* identified 7 functional SNPs associated with the development of OM in patients with nasopharyngeal carcinoma treated with RTH [15]. Also other researchers have confirmed the relationship between SNPs and radio-responsive genes or DNA DSB repair genes (Ku70 c.1781G> T) [21, 22].

There are few studies of the *APEH* gene. Wen *et al.* demonstrated that *APEH* polymorphism has significant influence on valproic acid pharmacokinetics in Chinese epileptic population [23]. Their participation in the development of inflammatory bowel disease and cancer cachexia was also studied [24, 25]. In a study of 1797 cancer patients, Solheim *et al.* described two SNPs from the *APEH* gene that showed a trend toward significance in association with cachexia [25]. However, there are no studies assessing the association of the *APEH* gene SNPs with OM.

Due to the fact that ROS play a key role in the pathomechanism of the development of OM, our study evaluated gene coding for the APEH enzyme, which takes an important part in their detoxification. We have determined the occurrence of *APEH* gene polymorphisms in HNC patients and showed that, from the second week of RTH, the CC genotype of the *APEH* gene (c.-521G> C, rs4855883) is an independent factor limiting the risk of OM intensification. The CC genotype is also an unfavorable prognostic factor for the OS.

To our knowledge, this is the first evaluation study using a large group of 62 patients irradiated for HNC. In these patients, the presence of *APEH* gene polymorphism is associated with OM severity and, concurrently, with OS.

The correlations we observed suggest that, in patients with the CC genotype of the *APEH* gene, the increased radiation resistance develops in a so far unknown mechanism. In result, severe OM in irradiated healthy tissue is observed less frequently but OS is lower.

The significantly lower incidence of OM observed in patients with the CC genotype of *APEH* gene may stem from many causes, because APEH may act directly or through the ubiquitin-proteasome system (UPS). In consequence, there are many biochemical pathways that can be modified when changing the function of the APEH enzyme [26–29].

Zeng *et al.* demonstrated that APEH is a component of cellular response to chromosomal DNA damage, following oxidative stress. They showed that APEH interacts directly with the DNA single-strand break repair scaffold protein XRCC1 and that this interaction mediates recruitment of APEH both into the nucleus and at sites of nuclear damage. Moreover, they show that in human cells APEH promotes both the repair of chromosomal single-strand breaks and cellular resistance to oxidative stress. These data identify APEH as a novel component of the DNA damage response, and the authors suggest that this protease facilitates protein metabolism at chromosomal sites of DNA strand breakage [26].

This action of the APEH enzyme is particularly important because DNA gets damaged in the first phase of OM [2]. Ionizing radiation used in RTH induces a plethora of DNA lesions, including oxidative base damage, as well as single and double-strand breaks. This leads to changes in structure and, ultimately, function of the DNA [30, 31]. Corrupted DNA of normal cells activates NF-κB and triggers a cascade of biochemical reactions responsible for the development of OM [2]. If cancer cells are able to efficiently repair the radiation damage, resistance to radiation develops, enabling cells to survive and replicate. If the damage remains unrepaired, these mechanisms induce programmed cell death or apoptosis [31].

It can not be ruled out that the presence of the CC genotype of the APEH gene may change its expression or function thus facilitate the repair of single and double-strand breaks caused by ionizing radiation. This would explain our observations that, in patients with the CC genotype, the lower incidence of serve OM is observed. In turn, facilitating the repair of DNA damage in tumor cells would lead to radiation resistance and, as a consequence, the higher risk of OS shortening observed by us.

At the final stage of OM progression, the so-called ulcerative phase, bacterial superinfection occurs. Cell wall products from bacteria can activate tissue macrophages, leading to increased production of the proinflammatory cytokines [2]. In the study on mouse model of lung inflammation, Komatsu *et al.* have demonstrated that the APEH activity in immune cells might determine the duration of inflammation induced by infection with different pathogens. They noticed that downregulation of APEH activity might result in more prolonged inflammation [27].

It cannot be ruled out that the significantly lower incidence severe OM observed in our study in patients with the CC genotype of the *APEH* gene results from the limitation of the inflammatory reaction caused by microorganisms in the ulcerative phase of OM.

One of the key functions of APEH is the active influence on the UPS [10, 11]. According to Palmieri *et al.*, despite the unknown mechanism of cooperation with UPS, APEH is a negative effector of proteasome activity [11]. By using a set of selected APEH inhibitors, they showed that proteasome activity can be regulated through an APEH-mediated mechanism [11].

It is known that UPS is a complex responsible primarily for the degradation of damaged proteins [28]. Thus, it participates in major intracellular processes, such as the cell cycle, cell differentiation, immune response, apoptosis [29]. Changing the UPS function through the existence of e.g. SNPs of the *APEH* gene may lead to slowing or inhibiting many biochemical reactions. This may be important in the development of OM and in the proliferation of tumor cells. Thus, the less frequent severe OM and lower OS rate in patients with the CC genotype of the *APEH* gene may indirectly result from the alteration of one of the metabolic pathways regulated by UPS.

Our study is the first to highlight the role of APEH in the pathogenesis of OM and OS prognosis. The CC genotype of the *APEH* gene characterizes patients with less severe OM but also lower OS.

Literature data confirm that anemia before the treatment and p16 expression are also significant prognostic factors in HNC patients [32–35]. However, the results of our study do not indicate hemoglobin level before RTH to have any influence on the survival of HNC patients. Neither has it demonstrated OM progression to be correlated with hemoglobin level before RTH. Such correlation was found by Becker-Schiebe *et al.* [36]. They confirmed that OM was observed less often in patients with pretreatment anemia, while p16 expression was significantly associated with severe OM [36]. Also a study by Hanasoge *et al.* conducted in p-16 positive patients demonstrated higher prevalence of OM [37]. These correlations may be explained by the fact that in the course of HPV infection the basal layer of squamous epithelium gets damaged, which leads to the intensification of radiation-related inflammation. In turn, tissue hypoxia caused by decreased hemoglobin values is one of the best understood factors affecting their radiosensitivity and responsiveness to ionizing radiation. The lack of correlation between hemoglobin level and OM in our study may be the result of small differences in the subjects’ hemoglobin levels.

Despite the not fully recognized mechanism responsible for up to 6-fold reduction in the occurrence of severe OM in patients with the CC genotype of the *APEH* gene, our study shows that APEH may play an important role in the development of OM. Simultaneously, reduction of OS in this group of patients suggests that the CC genotype may be an important prognostic factor. Due to the worse effects of treatment of patients with CC genotype of the *APEH* gene, there is a need to intensify treatment in this group of patients. It may be justified to use chemoradiation, immunotherapy, and, taking into account the low OM level, increasing the total dose of radiation or changing the dose fractionation. It can not be ruled out that the intensification of the treatment of patients with the genotype CC of the *APEH* gene will contribute to the improvement survival time of the patients.

## MATERIALS AND METHODS

### Patient and clinical data

62 HNC patients were enrolled in stages I-IV. Detailed clinical data and patient characteristics are presented in Table 1. The stage of disease was evaluated using VII-th edition of TNM classification (UICC). The level of alcohol consumption was evaluated using International Statistical Classification of Diseases and Related Health Problems (ICD). It was classified as either excessive (F10.1 and F10.2) or occasional. The follow-up time was 40 months. Pretreatment hemoglobin concentrations was checked 2–5 days prior to treatment. Anemia was defined as a hemoglobin level <12.0 g/dl.

The study design was approved by Bioethical Commission at the Medical University of Lublin (KE-0254/232/2014). All patients signed the informed consent form prior to the study.

### Radiotherapy

Shortly, all the patients were immobilized in supine position using a customized thermoplastic mask. CT imaging of the area of interest was performed for planning, with slice thickness of 3 mm. Bolus was not used. Our institutional treatment protocol was used to delineate the target volumes. The protocol conforms with the International Commission on Radiation Units and Measurements Reports 50 and 62.

IMRT plans were developed for all patients using Prowess Panther version 5.20 treatment planning system (Prowes, Inc., Concord, USA). Nine fixed-gantry angle coplanar beams with step-and-shoot treatment techniques on a linear accelerator (Siemens Artiste) were used in all plans. All patients were treated with 6-MV photons. In order to be approved the treatment plans had to meet the following criteria: (1) 95% of any planning target volume (PTV) at or above the prescribed dose; (2) 99% of any PTV at or above 90% of the PTV dose. Doses applied to organs at risk (OAR) were under the framework of Radiation Therapy Oncology Group (RTOG) 0225 protocol.

In patients with the history of surgical resection of tumor, clinical target volume (CTV) included surgical tumor bed with a margin of 1 cm and bilateral lymph nodes. Additional boost involving high risk area (positive margins, affected lymph nodes with extracapsular extension) was added. A PTV margin of 3 mm was added to CTV. The dose to CTV (60 Gy) and high risk (66 Gy) was planned.

In case of patients with gross lesion the prescribed dose of radiation was defined in the following manner: a total dose of 54 Gy to low-risk targets (CTV 54), 60 Gy to the entire anatomical subsite and the affected lymph nodes (CTV 60) and 70 Gy to gross tumor volume with 1 cm margin (CTV 70). A PTV margin of 3 mm was added to CTV54, CTV 60 and CTV 70. The treatment was delivered once daily, over 5 fractions per week. 58 patients completed the prescribed dose of IMRT without interruption.

The PF scheme administered in neoadjuvant treatment CTH was: cisplatin (100 mg/m^2^ on day 1) and 5-FU (1,000 mg/m^2^ per day, continuous infusion on days 1–5) in 21-day cycles. In the course of concurrent chemoradiation, cisplatin was administered in the dose of 100 mg/m^2^ every 21 days.

### The assessment of toxicity

The OM intensity was evaluated using RTOG/EORTC scale. The assessment was carried out at baseline and every week of treatment afterwards. Upon the end of RTH, the patients were assessed at 6-week intervals in the first year, then every 3 months for the next 2 years, and every 6 months thereafter.

### Genotyping

The isolation of DNA from peripheral blood was performed using DNA Blood Mini Kit (Qiagen, Canada). Quality and quantity measurements of the obtained RNA were performed using NanoDrop2000c (Thermo Scientific) spectrophotometer. The analysis of *APEH* SNP was carried out using minisequencing technique (SNaPshot^®^ PCR). ABI PRISM SNaPshot^®^ Multiplex (Life Technologies, USA) kit was used to carry out the SNaPshot^®^ PCR reaction.

We have validated results of allelic discrimination of our method (SNaPhot PCR) by performing another analysis of a studied SNP in a different set of patients (*n* = 26) and we obtained a similar distribution of genotypes (CC - 30.8%, CG - 57.7%, GG - 11.5%). Moreover, in the validation group, we obtained the same results of genotype frequencies (full compliance) when the different genotyping method (allele-specific PCR) was used.

### Statistical analysis

The results for the analyzed gene genotyping were retrospectively linked with the patients’ OM and OS. The obtained results were statistically analyzed using MedCalc15.8 Software (Belgium). The result values of *p* < 0.05 were regarded as statistically significant. Chi Square (χ^2^) test was used to evaluate Hardy-Weinberg (H-W) balance, the occurrence and intensity of OM and the correlation between several demographic-clinical factors. Odds Ratio (OR) was used to evaluate the risk of OM development in relation to demographic-clinical factors distribution and polymorphic variants of *APEH* gene. The Kaplan-Meier method and Cox regression analysis were used to assess the probability of OS in relation to *APEH* genotype.
